# Hepatic Encephalopathy Induced by Small Bowel Obstruction in a Patient With Primary Biliary Cholangitis

**DOI:** 10.7759/cureus.87169

**Published:** 2025-07-02

**Authors:** Alayobi Elbasheer, Salah Mohamed Magzoub Ahmed

**Affiliations:** 1 Internal Medicine, Oxford University Hospitals NHS Foundation Trust, Oxford, GBR; 2 Gastroenterology, Betsi Cadwaladr University Health Board, Bangor, GBR

**Keywords:** ammonia, hepatic encephalopathy, primary biliary cholangitis, rifaximin, small bowel obstruction

## Abstract

Hepatic encephalopathy (HE) represents a spectrum of neuropsychiatric abnormalities resulting from hepatic dysfunction. This case report discusses the case of a 66-year-old woman with a known diagnosis of primary biliary cholangitis (PBC) who presented with a 10-day history of constipation, vomiting, and progressive cognitive decline. Cross-sectional imaging of the abdomen revealed a large ventral hernia in the anterior midline containing multiple loops of small bowel, consistent with adhesional small bowel obstruction. Laboratory investigations demonstrated elevated ammonia levels and deranged liver function tests (LFTs), consistent with the clinical picture of HE. The patient was managed conservatively with bowel decompression, and HE was successfully treated with rifaximin and, following the resolution of the obstruction, with lactulose. The patient made a good neurological recovery with normalization of biochemical markers and was discharged from the intensive care unit (ICU) in a stable condition. This case underscores the importance of recognizing and promptly addressing reversible precipitants of HE and illustrates the challenges of managing HE in the context of gastrointestinal obstruction.

## Introduction

Hepatic encephalopathy (HE) is commonly observed in patients with cirrhosis and manifests as a spectrum of neuropsychiatric abnormalities, ranging from subtle cognitive changes and sleep disturbances to severe disorientation, altered consciousness, and coma. It can be explained, to some extent, by the effect of neurotoxic substances that accumulate in the setting of acute or chronic liver disease [1]. Liver cirrhosis signifies irreversible liver damage and the loss of liver architecture due to fibrosis, leading to impaired detoxification [2].

As liver decompensation occurs, nitrogenous wastes like ammonia accumulate and cross the blood-brain barrier (BBB), where they are taken up by astrocytes. Inside these cells, glutamate is metabolized into glutamine. The excess glutamine causes an osmotic imbalance, resulting in an intracellular shift of fluid and subsequent cerebral edema. The clinical severity of HE is graded on a scale from one to four [2]: (i) Grade 1 representing altered mood/behaviour, sleep disturbance (reversal of sleep pattern), without asterixis; (ii) Grade 2 representing increased drowsiness, confusion, slurred speech, asterixis, and personality changes; (iii) Grade 3 representing incoherent speech, stupor, restlessness, and asterixis; and (iv) Grade 4 representing coma.

Primary biliary cholangitis (PBC), previously called primary biliary cirrhosis, is a progressive autoimmune cholestatic liver disease characterized by destruction of the interlobular bile duct [3]. While not all PBC patients have cirrhosis, hepatic decompensation and its complications are more common in the late stages of the disease.

In the setting of liver disease, HE can be triggered by several factors, including electrolyte imbalances, infections, delayed gastrointestinal transit, and gastrointestinal bleeding [4]. Small bowel obstruction can lead to delayed ammonia clearance, thereby increasing systemic ammonia levels and precipitating HE. This case report discusses the clinical course and management of such a case and highlights the importance of identifying and treating underlying triggers.

## Case presentation

A 66-year-old woman presented to the emergency department with a 10-day history of constipation despite using suppositories, persistent vomiting, decreased oral intake, and progressive confusion. She was afebrile on presentation and denied any episodes of per rectal bleeding. Of note, she had recently commenced a course of acyclovir for shingles affecting the left L1 dermatome.

Her past medical history included hypertension, stage 3 chronic kidney disease, PBC with anti-mitochondrial antibody (AMA) positivity, and she was taking ursodeoxycholic acid (UDCA). There was no previous imaging indicating cirrhosis. She had undergone a total abdominal hysterectomy for a mid-pelvic mass and had a prior diagnosis of Hodgkin’s lymphoma, for which she was not receiving chemotherapy due to recurrent episodes of neutropenic sepsis. 

On arrival, her observations were within normal limits; however, she had overtly depressed consciousness with a Glasgow Coma Scale (GCS) score of 7. Physical examination revealed a distended but soft, non-tender abdomen, along with the presence of an irreducible, non-tender hernia in the lower periumbilical area.

Within hours of admission, her neurological status deteriorated to a GCS score of 4, despite stable hemodynamic parameters. Her neurological deterioration was accompanied by an episode of transient clonic jerking in the upper limbs, thought to be related to her evolving HE.

Investigations

Initial laboratory investigations revealed an acute-on-chronic kidney injury with a drop in estimated glomerular filtration rate (eGFR) from a baseline of 38 to 24, elevated inflammatory markers, and deranged liver biochemistry with significantly raised ammonia levels.

In light of her low GCS score, she was admitted to the critical care unit, and further investigations revealed an ammonia level of 426 umol/L. A full list of admission investigations is demonstrated in Table 1.

**Table 1:** Investigations on admission WBC: White blood cell count, Hb: Hemoglobin, eGFR: Estimated glomerular filtration rate, ALP: Alkaline phosphatase, GGTP: Gamma-glutamyltransferase

Laboratory investigation	Initial value	Normal value
WBC	13 x 10^9^/L	3.7-11 x 10^9^/L
Hb	177 g/L	120-165 g/L
Urea	20.4 mmol/L	2.5-7.8 mmol/L
Serum creatinine	182 umol/L	49-90 umol/L
eGFR	24 mL/min/1.73 m²	>90 mL/min/1.73 m²
Lactate	3.7 mmol/L	0.6-2 mmol/L
Total bilirubin	40 umol/L	0-21 umol/L
ALP	146 U/L	30-130 U/L
GGTP	106 U/L	0-38 U/L
Ammonia	426 umol/L	<50 umol/L

**Figure 1:**
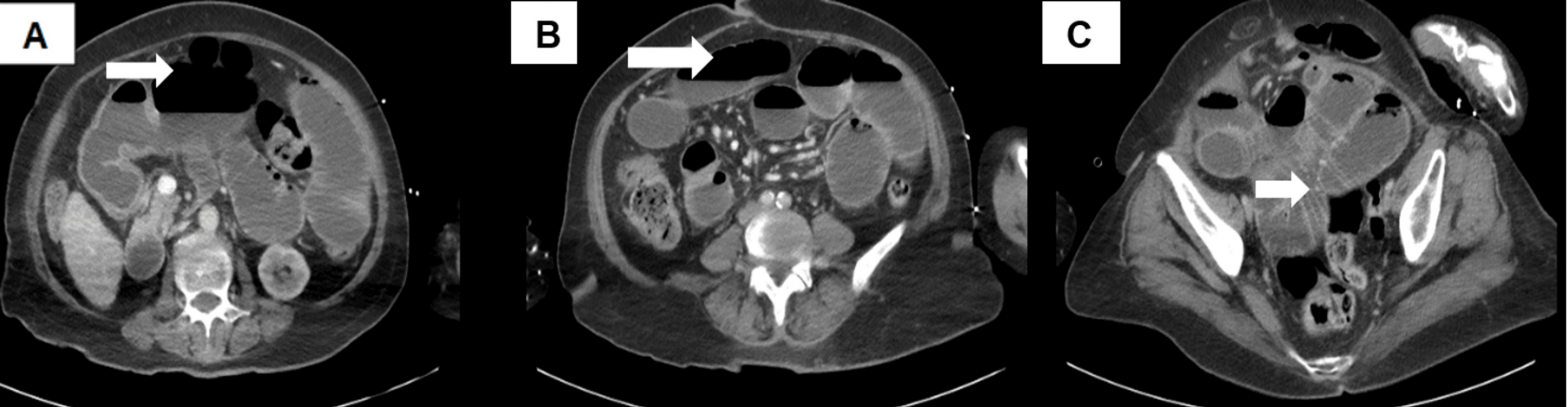
CT scan on admission (A, B) demonstrate dilated small bowel with an air-fluid level. (C) demonstrates adhesions within the small bowel. CT: Computed tomography

Cross-sectional imaging included a computed tomography (CT) scan of the brain, which revealed no acute intracranial pathology to explain the drop in cognition. Given the clinical severity and diagnostic uncertainty, CT imaging of the thorax, abdomen, and pelvis (Figure 1) was also performed to further evaluate the gastrointestinal findings and underlying biochemical derangement. The images demonstrated features consistent with small bowel obstruction, likely secondary to adhesions involving loops of bowel within a midline ventral hernia. No evidence of ascites was noted on imaging.

Differential diagnosis

Given the acute decline in her level of consciousness, the initial differential included viral encephalitis, particularly herpes zoster encephalitis related to her recent shingles infection, as well as meningoencephalitis. However, in the context of markedly deranged liver function tests (LFTs), particularly elevated ammonia levels, the patient was diagnosed as likely metabolic encephalopathy with possible superimposed infection of unclear etiology, and empirical therapy was initiated.

An electroencephalogram (EEG) was initially requested to evaluate for periodic lateralized epileptiform discharges (PLEDs) and support the diagnosis of metabolic encephalopathy. This was ultimately not performed, as the patient's neurological status improved significantly with medical management.

Treatment

Following the working diagnosis of HE with a possible superimposed infection, the patient was transferred to the intensive care unit (ICU) and intubated for airway protection due to her significantly reduced GCS. Surgical review suggested a conservative approach to the small bowel obstruction given her comorbidities and high perioperative risk. Nasogastric decompression via Ryle’s tube (Pennine Healthcare, UK) was initiated. She was started on rifaximin, and once the obstruction resolved, she was commenced on lactulose. Empiric antibiotics with intravenous acyclovir and ceftriaxone were administered to cover for potential meningoencephalitis and secondary infection.

The patient’s clinical condition initially improved with the above regimen, and ammonia levels declined significantly, correlating with neurological recovery. LFTs normalized, including bilirubin levels. The patient was gradually weaned off ventilatory support and discharged from the ICU in a stable condition on day six of the admission.

Outcome and follow-up

An inpatient FibroScan (Echosens, France) was performed, revealing stiffness suggestive of a cirrhotic liver: 43.3 kpa, interquartile range (IQR) 10.3, IQR medium 24%, controlled attenuation parameter (CAP) 219. During her continued inpatient stay, the patient was assisted with inpatient rehabilitation, dietetic support, and speech therapy to aid functional recovery. She was ultimately discharged back to her own home with a planned outpatient hepatology follow-up.

## Discussion

HE is defined as a brain dysfunction caused by hepatic insufficiency and/or portosystemic shunting. Mild subclinical changes may include inversion of the sleep cycle and progress to marked disorientation and coma [5]. This is thought to be due to the accumulation of encephalopathic waste products, including ammonia, normally metabolised in the liver [6]. In the setting of encephalopathy, these materials bypass the liver (portosystemic shunting) or are ineffectively metabolised by a failing liver and cross the BBB, ultimately causing neuropsychiatric symptoms. It is important to note, however, that HE is not exclusive to patients with cirrhosis but may also occur in the context of non-cirrhotic portal hypertension [7].

This case report discusses this phenomenon in a patient with known liver pathology (PBC). Although not all patients with PBC develop cirrhosis, advanced cases like this one can cause significant hepatic dysfunction and predispose to encephalopathy. In this case, the triggering factor was constipation due to small bowel obstruction.

Similar mechanisms have been reported in both pediatric and adult patients. For instance, Gonzales et al. described a case involving a non-cirrhotic child with a mesocaval shunt who developed HE following small bowel obstruction [8]. In this case, adhesiolysis relieved the obstruction and resulted in the resolution of the encephalopathy. Kawakami et al. described the case of a 79-year-old woman with status epilepticus secondary to severe hyperammonaemia precipitated by large bowel obstruction in the setting of a congenital portosystemic shunt [9]. In this case, surgical decompression via colostomy led to rapid biochemical and clinical improvement.

At present, in the UK, according to the National Institute for Health and Care Excellence (NICE) guidelines, treatment of HE is directed primarily to reduce the production and absorption of ammonia. This is typically achieved through bowel cleansing with non-absorbable disaccharides like lactulose and the use of non-absorbable antibiotics like rifaximin to prevent recurrence of overt HE [10,11].

The use of rifaximin in the treatment of HE is well-documented, given that it has very low bioavailability and local action within the gut when taken orally due to its poor enteral absorption [10]. This case required a modified approach due to the presence of small bowel obstruction, which contraindicated the use of lactulose. Without active gut transit, lactulose would have been ineffective and potentially harmful. This approach aligns with other reports, such as the case by Ustaoglu et al., where hyperammonaemia due to vomiting-induced hypokalaemia in the context of bowel obstruction resolved with potassium correction and reduction of the hernia [12]. Another case reported the use of neostigmine to initiate peristaltic movement in a cirrhotic patient with HE and acute intestinal pseudo-obstruction [13].

In cirrhotic patients, abdominal wall hernias are common due to prolonged intra-abdominal pressure from ascites and malnutrition [14]. While this patient did not have overt ascites, the presence of a ventral hernia was likely attributable to prior abdominal surgery, hypalbuminaemia, and malnutrition. The subsequent small bowel obstruction was a direct trigger for decompensation. Surgical correction of abdominal wall hernias in cirrhotic patients does not occur frequently due to the associated perioperative complications and mortality [15].

Lastly, while the clinical presentation was most consistent with HE, other differential diagnoses were considered. As NICE highlights, the diagnostic process in encephalopathy should include exclusion of mimics such as electrolyte disturbances, infections, and structural brain lesions [5]. However, in this case, the temporal association between resolution of the bowel obstruction and neurological recovery strongly supported a primary diagnosis of HE precipitated by intestinal stasis.

## Conclusions

This case exemplifies the stigmata associated with HE, driven by neurotoxicity from accumulated metabolic toxins. Effective management requires prompt identification or exclusion of triggers, and while a contribution from viral encephalitis or delirium could not be entirely excluded, both were empirically treated. In this case, constipation secondary to adhesional small bowel obstruction was the primary trigger. The close temporal link between the resolution of bowel obstruction and neurological recovery strongly supported HE precipitated by intestinal stasis. Treatment focused on bowel cleansing, with lactulose enhancing ammonia excretion and rifaximin reducing its production. Optimal outcomes in HE require prompt intervention, coordinated multidisciplinary care, and continuous assessment for reversible triggers.
